# *KCNJ5* Somatic Mutations in Aldosterone-Producing Adenoma Are Associated with a Greater Recovery of Arterial Stiffness

**DOI:** 10.3390/cancers13174313

**Published:** 2021-08-26

**Authors:** Yi-Yao Chang, Chien-Ting Pan, Zheng-Wei Chen, Cheng-Hsuan Tsai, Shih-Yuan Peng, Chin-Chen Chang, Bo-Ching Lee, Che-Wei Liao, Kang-Yung Peng, Yu-Wei Chiu, Chia-Hung Chou, Vin-Cent Wu, Li-Yu Daisy Liu, Chi-Sheng Hung, Yen-Hung Lin

**Affiliations:** 1 National Taiwan University College of Medicine Graduate Institute of Clinical Medicine, Taipei 100, Taiwan; rollerpapa@mail.chihlee.edu.tw; 2 Cardiology Division of Cardiovascular Medical Center, Far Eastern Memorial Hospital, New Taipei City 220, Taiwan; dtmed005@saturn.yzu.edu.tw; 3 Division of Cardiology, Department of Internal Medicine, National Taiwan University Hospital and National Taiwan University College of Medicine, Taipei 100, Taiwan; d09421005@ntu.edu.tw (C.-H.T.); sypeng@ntu.edu.tw (S.-Y.P.); kangyung@ntu.edu.tw (K.-Y.P.); 4 Center of General Education, Chihlee University of Technology, New Taipei City 220, Taiwan; 5 Department of Internal Medicine, National Taiwan University Hospital Yun-Lin Branch, Yun-Lin 640, Taiwan; y04444@ms1.ylh.gov.tw (C.-T.P.); librajohn7@hotmail.com (Z.-W.C.); 6 Department of Internal Medicine, National Taiwan University Hospital Jin-Shan Branch, New Taipei City 220, Taiwan; 7 Department of Medical Imaging, National Taiwan University Hospital and National Taiwan University College of Medicine, Taipei 100, Taiwan; ccchang@ntuh.gov.tw (C.-C.C.); bclee@ntuh.gov.tw (B.-C.L.); 8 Department of Medicine, National Taiwan University Cancer Center, Taipei 106, Taiwan; A01217@ntucc.gov.tw; 9 Department of Computer Science and Engineering, Yuan Ze University, Taoyuan City 320, Taiwan; 10 Department of Obstetrics and Gynecology, National Taiwan University Hospital and National Taiwan University College of Medicine, Taipei 100, Taiwan; d03421008@ntu.edu.tw; 11 Division of Nephrology, Department of Internal Medicine, National Taiwan University Hospital and National Taiwan University College of Medicine, Taipei 100, Taiwan; q91421028@ntu.edu.tw; 12 Department of Agronomy, Biometry Division, National Taiwan University, Taipei 106, Taiwan; lyliu@ntu.edu.tw; 13 Cardiovascular Center, National Taiwan University Hospital, Taipei 100, Taiwan

**Keywords:** *KCNJ5* somatic mutation, pulse wave velocity, aldosterone-producing adenoma, adrenalectomy, propensity score matching, arterial stiffness

## Abstract

**Simple Summary:**

Primary aldosteronism (PA) is the most common form of secondary hypertension and induces various cardiovascular injuries. Aldosterone-producing adenoma (APA) is one of the major forms of PA. The occurrence of APA is closely correlated with somatic mutations, including *KCNJ5.* We described here the impact of *KCNJ5* somatic mutations on arterial stiffness excluding the influence of age, sex, and blood pressure status. We found *KCNJ5* mutation carriers had similar arterial stiffness before surgery, but greater improvement of arterial stiffness after adrenalectomy compared with non-carriers. Hence, APA patients with *KCNJ5* mutations had a greater improvement in arterial stiffness after adrenalectomy than those without mutations.

**Abstract:**

Primary aldosteronism is the most common form of secondary hypertension and induces various cardiovascular injuries. In aldosterone-producing adenoma (APA), the impact of *KCNJ5* somatic mutations on arterial stiffness excluding the influence of confounding factors is uncertain. We enrolled 213 APA patients who were scheduled to undergo adrenalectomy. *KCNJ5* gene sequencing of APA was performed. After propensity score matching (PSM) for age, sex, body mass index, blood pressure, number of hypertensive medications, and hypertension duration, there were 66 patients in each group with and without *KCNJ5* mutations. The mutation carriers had a higher aldosterone level and lower log transformed brachial–ankle pulse wave velocity (baPWV) than the non-carriers before PSM, but no difference in log baPWV after PSM. One year after adrenalectomy, the mutation carriers had greater decreases in log plasma aldosterone concentration, log aldosterone–renin activity ratio, and log baPWV than the non-carriers after PSM. Only the mutation carriers had a significant decrease in log baPWV after surgery both before and after PSM. *KCNJ5* mutations were not correlated with baseline baPWV after PSM but were significantly correlated with ∆baPWV after surgery both before and after PSM. Conclusively, APA patients with *KCNJ5* mutations had a greater regression in arterial stiffness after adrenalectomy than those without mutations.

## 1. Introduction

Primary aldosteronism (PA) is the most common form of secondary endocrine hypertension, which accounts for 5–15% of all cases of hypertension [1,2,3]. Excessive aldosterone results in various vascular structure injuries. Previous animal studies have shown that aldosterone infusion in uninephrectomized rats accompanied with a high sodium diet could cause increased arterial stiffness associated with fibronectin accumulation [4]. In addition, this effect was independent of wall stress as shown by normotensive controls and reversal of vascular damage by treatment with an aldosterone antagonist [4]. In clinical studies, increased carotid–femoral pulse wave velocity (PWV), which represents increased arterial stiffness, has been noted in patients with PA compared to patients with essential hypertension (EH) after adjusting for all clinical variables including 24 h blood pressure [5]. These effects on arteries have been shown to be reversed after adrenalectomy [6,7].

Aldosterone-producing adenoma (APA) is one of the major subtypes of PA, and it can be cured by adrenalectomy [8,9]. Channelopathies resulting from somatic mutations have been identified as the main pathogenesis of APA in recent years [10,11,12,13,14]. Mutations of the *KCNJ5* gene (coding for G protein-activated inward rectifier potassium channels [10]) are the most common, with a prevalence rate of around 40% in Western countries [15,16,17] but 55–75% in Asian countries [18,19,20,21]. Some common clinical phenotypes in both Western and Asian APA patients with *KCNJ5* mutations have been observed, including younger age, higher aldosterone level, lower potassium level, and higher hypertension cure rate [15,16,17,22]. However, differences in sex and tumor size have not been found in most Asian studies [20,23,24].

Previous studies have reported associations between *KCNJ5* somatic mutations and worse left ventricular remodeling but better recovery after adrenalectomy [22,25]. However, only a few studies have investigated the impact of *KCNJ5* somatic mutations on arterial stiffness. Previous studies have reported a lower PWV in APA patients with *KCNJ5* mutations compared to those without mutations [23,24]. However, a younger age in patients with mutations would cause a lower PWV, which would then interfere with the interpretation of the effect of *KCNJ5* mutations. In contrast, an earlier study from our group showed that APA patients with *KCNJ5* mutations had comparable PWV to patients without *KCNJ5* mutations both before and after matching for age, sex, and body mass index [26]. In addition, the influence of *KCNJ5* mutations on the change in PWV after adrenalectomy is still unclear.

This study was designed to investigate the role of *KCNJ5* mutations on arterial stiffness and its reversal after adrenalectomy. We used propensity score matching (PSM) analysis to attenuate possible confounding factors.

## 2. Materials and Methods

### 2.1. Patients

In this prospective study, we enrolled 213 APA patients who were scheduled to undergo adrenalectomy from January 2007 to May 2019 at National Taiwan University Hospital. The medical histories, including demographic data, severity of blood pressure, and medications of all patients were reviewed carefully. Serum biochemical and brachial–ankle pulse wave velocity (baPWV) data were acquired at the initial evaluation of the patients, and again 1 year after adrenalectomy. Cure was defined as patients who had normalized blood pressure (systolic blood pressure (SBP) < 140 mmHg and diastolic blood pressure (DBP) < 90 mmHg) independently of any antihypertensive drugs after adrenalectomy, which is the same as the definition of “completely clinically cured” proposed by the Primary Aldosteronism Surgical Outcomes (PASO) group [27]. Informed consent was obtained from all patients prior to inclusion in the study, and the study was approved by the Ethics Committee of National Taiwan University Hospital (approval number: 200611031R).

### 2.2. Laboratory Measurements

Plasma aldosterone concentration (PAC) was measured using a commercial radioimmune assay kit (Aldosterone Maia Kit; Adaltis Italia, Bologna, Italy). Plasma renin activity (PRA) was measured according to the generation of angiotensin-I in vitro using a commercial radioimmune assay kit (DiaSorin, Stillwater, MN, USA). The aldosterone-to-renin ratio (ARR) was calculated as the PAC divided by the PRA.

### 2.3. Diagnostic Criteria for Aldosterone-Producing Adenomas

The diagnosis of APA was confirmed according to the consensus of the Taiwan Society of Aldosteronism [28] and “modified four corner criteria” after adrenalectomy [29,30] as follows: (1) excess aldosterone production in accordance with an ARR > 35, TAIPAI score > 60% [31], and seated post-saline loading PAC > 16 ng/dL or urine aldosterone > 12 μg/24 h [32]; (2) adenoma on a CT scan; (3) lateralization of excessive aldosterone secretion according to adrenal venous sampling or dexamethasone suppression NP-59 single-photon emission computed tomography (SPECT/CT) [33]; (4) adenoma in histopathological analysis after adrenalectomy, and subsequently either a biochemical cure pattern with improvement of hypertension or clinical cure pattern of hypertension without antihypertensive drugs.

### 2.4. Arterial Stiffness Evaluation

We measured the baPWV of the patients in a supine position with an autonomic waveform analyzer (Colin VP-2000, Omron Inc., Kyoto, Japan) after a rest of at least 15 min. The analyzer recorded bilateral brachial and tibial arterial pressure waveforms, phonocardiogram, and electrocardiogram. The time difference from brachial to ankle arterial pressure wave was determined according to the wave front velocity theory [34]. The distance between arm and ankle was expressed as a linear equation of height. The baPWV was calculated as the distance divided by the time difference. Finally, the average of right and left baPWV values in each patient was used for further analysis.

### 2.5. Adrenalectomy

All of the APA patients underwent laparoscopic adrenalectomy via a lateral trans-peritoneal approach by experienced laparoscopic surgeons.

### 2.6. Histopathologic Studies

Adrenal specimens were blindly assessed by an experienced pathologist. Nodules comprised of adrenal cortical cells and clearly demarcated by a pseudo-capsule were defined as adenomas [35]. Adenomas were differentiated from nodular hyperplasia if they were isolated and well-circumscribed [36].

### 2.7. Genomic DNA Extraction and Sequencing of the KCNJ5 Gene

Adrenal specimens were stored at −80 °C after adrenalectomy. Genomic DNA was extracted using a QIAamp DNA mini kit (Qiagen, Hilden, Germany) from 213 peritumoral adrenal cortices.

We analyzed the coding regions of the genomic DNA via exome sequencing. The entire coding sequence (exons 2–3) and flanking regions of the *KCNJ5* gene were amplified and sequenced using four sets of gene-specific primers as reported previously [37] (listed in Table S1). GoTaq^®^ Master Mix (Promega Corporation, Madison, WI, USA) was used for the PCR reactions with an annealing temperature of 58 °C. GenepHlow™ Gel/PCR Kits (Geneaid, Taipei, Taiwan ROC) were used to extract DNA fragments from PCR. Sanger sequencing of PCR products was carried out using a 3730 DNA Analyzer (Applied Biosystems, Foster City, CA, USA).

### 2.8. Statistical Analysis

SPSS for Windows version 25.0 (SPSS Inc., Chicago, IL, USA) with the R-3.3 plugin extension was used for the propensity score matching (PSM) analysis. A 1:1 matching ratio was adopted. Propensity scores were assessed using a non-parsimonious multiple logistic regression model including the following probable confounding variables in patients with and without *KCNJ5* somatic mutations: age, sex, body mass index (BMI), SBP, DBP, number of hypertensive medications, and duration of hypertension. The balance of the selected covariates for matching between the matched groups was subsequently examined.

All continuous variables were presented as mean ± SD. Non-normally distributed variables were presented as median and interquartile range, including PAC, PRA, and ARR. The equality of two proportions was evaluated using the Pearson chi-square test. Comparisons of continuous data between two groups were conducted using the Student’s t test for normally distributed variables or the Wilcoxon rank-sum test for non-normally distributed variables. Comparisons of continuous data before and after adrenalectomy were performed using paired t tests. PAC, PRA, and ARR data were log-transformed due to non-normal distribution as determined by the Kolmogorov–Smirnov test for further regression analysis. Correlations of *KCNJ5* mutations with baseline log baPWV and the change in log baPWV after adrenalectomy before and after PSM were analyzed using linear regression analysis with different adjustment models.

## 3. Results

### 3.1. Clinical and Biochemical Data of All APA Patients before and after Matching

Of the 213 APA patients who received adrenalectomy, 126 (59.2%) had *KCNJ5* somatic mutations. Of these 126 mutation carriers, sequencing of adenoma specimens demonstrated that 75 patients had p.Gly151Arg (c.451G > A or c.451G > C), 45 had p.Leu168Arg (c.503T > G), 3 had p.Thr158Ala (c.472A > G), and 3 had p.Glu145Gln (c.433G > C) mutations in the heterozygous state.

The *KCNJ5* mutation carriers were younger (*p* < 0.001), had a shorter duration of hypertension (*p* = 0.018), higher DBP (*p* = 0.003), higher aldosterone level (*p* < 0.001), higher ARR (*p* = 0.003), and lower potassium level (*p* < 0.001) (Table 1).

After 1:1 PSM for age, sex, BMI, SBP, DBP, duration of hypertension, and number of hypertensive medications, there were 66 patients in each group (*KCNJ5* mutation carrier group and non-carrier group). The matched APA patients with *KCNJ5* mutations had a lower rate of angiotensin-converting enzyme inhibitor (ACEI) or angiotensin II blocker (ARB) use (*p* = 0.037), higher aldosterone level (*p* = 0.012), higher ARR (*p* = 0.017), and lower potassium level (*p* < 0.001) than the non-carriers (Table 1).

### 3.2. baPWV of All APA Patients before and after Matching

Before PSM, the patients with *KCNJ5* mutations had a lower log baPWV (*p* = 0.046) compared to the non-carriers (Table 1). After PSM, there was no significant difference in log baPWV between the two groups (Table 1).

### 3.3. The Change in Clinical Data after Adrenalectomy before and after Matching

One year after adrenalectomy, the APA patients with *KCNJ5* mutations had a significantly higher cure rate (79% vs. 61%, *p* = 0.004) before PSM, and borderline higher cure rate (79% vs. 64%, *p* = 0.055) after PSM.

Before PSM, the APA patients with *KCNJ5* mutations had a greater decrease in SBP (*p* = 0.002), DBP (*p* = 0.003), number of hypertensive drugs (*p* = 0.001), log PAC (*p* < 0.001), log PRA (*p* = 0.034), and log ARR (*p* = 0.001), and greater increase in creatinine (*p* = 0.009) and potassium (*p* < 0.001) compared to the patients without *KCNJ5* mutations (Table 2). After PSM, the decrease in log PAC (*p* = 0.033) and log ARR (*p* = 0.015) and increase in potassium (*p* < 0.001) were still significantly higher in the matched APA patients with *KCNJ5* mutations than in those without *KCNJ5* mutations (Table 2).

**Figure 1:**
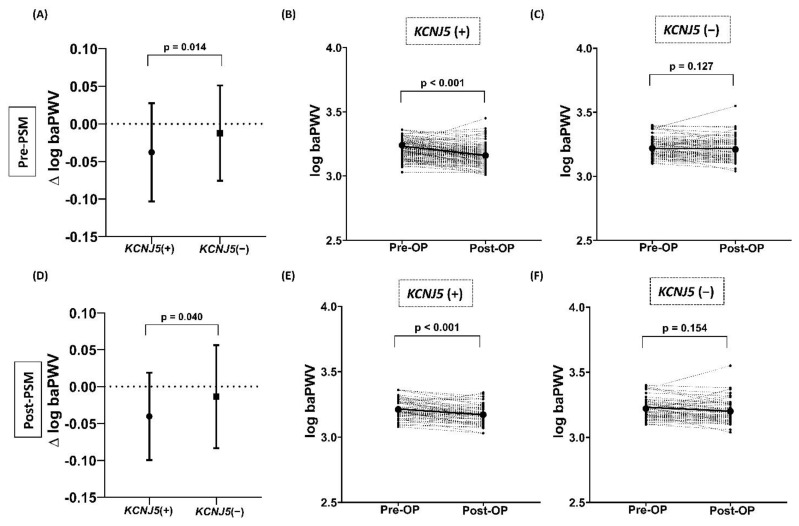
The changes in log baPWV after adrenalectomy between the APA patients with and without *KCNJ5* mutations before and after PSM. (**A**) Before PSM, the decrease in log baPWV after adrenalectomy was significantly greater in the patients with *KCNJ5* mutations than in those without mutations. (**D**) Even after PSM, the decrease in log baPWV after adrenalectomy was still significantly greater in the patients with *KCNJ5* mutations than in those without mutations. (**B**,**C**) Before PSM, only the patients with *KCNJ5* mutations had a significant decrease in log baPWV, and this was not seen in the patients without *KCNJ5* mutations. (**E**,**F**) After PSM, still only the patients with *KCNJ5* mutations had a significant decrease in log baPWV, and again this was not seen in the patients without *KCNJ5* mutations. APA, aldosterone-producing adenoma; baPWV, brachial–ankle pulse wave velocity.

For the change in log baPWV, the APA patients with *KCNJ5* mutations had a significantly greater decrease than the patients without *KCNJ5* mutations both before (*p* = 0.014) and after (*p* = 0.040) PSM (Figure 1A,D).

### 3.4. Paired Comparisons of Clinical Data in All Patients before PSM before and after Adrenalectomy, and Comparisons of Parameters 1 Year after Surgery between the APA Patients with and without Mutations

Before PSM, both the APA patients with and without *KCNJ5* mutations had significant decreases in SBP, DBP, number of hypertensive drugs, and log ARR, and both groups had significant increases in creatinine, potassium, and log PRA after adrenalectomy (Table 3). However, only the APA patients with *KCNJ5* mutations had a significant decrease in log PAC (*p* < 0.001) and log baPWV (*p* < 0.001) after adrenalectomy, which was not found in the patients without *KCNJ5* mutations (Figure 1B,C).

In addition, the patients with *KCNJ5* mutations had a significantly lower SBP (*p* = 0.044), number of hypertensive drugs (*p* = 0.010), and log baPWV (*p* < 0.001) 1 year after adrenalectomy compared to those without mutations.

### 3.5. Paired Comparisons of Clinical Data in Matched Patients before and after Adrenalectomy

After PSM, both the APA patients with and without *KCNJ5* mutations had significant decreases in SBP, DBP, number of hypertensive drugs, and log ARR, and both groups had significant increases in potassium and log PRA after adrenalectomy (Table 4). However, only the APA patients with *KCNJ5* mutations had a significant increase in creatinine (*p* = 0.001) and decrease in log PAC (*p* < 0.001) and log baPWV (*p* < 0.001) after adrenalectomy, which was not found in the patients without *KCNJ5* mutations (Figure 1E,F).

In addition, the matched patients with *KCNJ5* mutations had a significantly higher number of hypertensive drugs (*p* = 0.013), and log baPWV (*p* = 0.045) 1 year after adrenalectomy compared to those without mutations.

### 3.6. Correlation of KCNJ5 Mutations with Baseline log baPWV and the Change in log baPWV before and after PSM

Before PSM, the patients with *KCNJ5* mutations were correlated with baseline log PWV in Model 1 analysis (*p* = 0.046, without adjustments) (Table 5). However, after adjusting for age and sex (Model 2 analysis), the correlation between *KCNJ5* mutations and baseline log PWV was no longer significant. This was also found in subsequent analysis models. In contrast, the patients with *KCNJ5* mutations were correlated with the change in log PWV in all of the analysis models, including unadjusted (Model 1, *p* = 0.014), adjusted for age and sex (Model 2, *p* = 0.017), adjusted for age, sex, SBP, and DBP (Model 3, *p* = 0.043), and adjusted for age, sex, SBP, DBP, hypertensive drugs, and hypertension duration (Model 4, *p* = 0.039).

After PSM, the patients with *KCNJ5* mutations were not correlated with baseline log PWV in any of the analysis models (Table 5). In contrast, the patients with *KCNJ5* mutations were correlated with the change in log PWV in all of the analysis models, including unadjusted (Model 1, *p* = 0.040), adjusted for age and sex (Model 2, *p* = 0.049), adjusted for age, sex, SBP, and DBP (Model 3, *p* = 0.036), and adjusted for age, sex, SBP, DBP, hypertensive drugs, and hypertension duration (Model 4, *p* = 0.043).

## 4. Discussion

The major findings of this study were as follows. First, the APA patients with *KCNJ5* mutations had lower baseline baPWV compared to those without mutations; however, there was no difference after matching for age, sex, and blood pressure. Second, after adrenalectomy, the patients with *KCNJ5* mutations had a greater decrease in baPWV compared to those without mutations both before and after matching. Third, only the patients with *KCNJ5* mutations had a significant improvement in baPWV after adrenalectomy, and this was not seen in those without mutations either before or after matching. Finally, *KCNJ5* mutations were correlated with the change in baPWV even after adjusting for age, sex, and baseline blood pressure status both before and after matching.

Arterial stiffness can be caused by various etiologies, including age, hypertension, and hyperglycemia. PWV is a global cardiovascular indicator of arterial stiffness [38]. A pulse wave is produced from the ejection of blood from the heart. PWV is the speed of a pulse wave propagating to the periphery and is calculated as the distance of a pulse wave travelled divided by the time difference [39]. baPWV was developed around 20 years ago, and it is widely used to measure PWV due to its simplicity, convenience, and reliable reproducibility, especially in Japan and Asian countries [40,41]. Recent studies have shown that baPWV is a good predictor of cardiovascular events [42,43,44,45]. In a meta-analysis including 18 studies [43], Vlachopoulos et al. reported that an increase in baPWV of 1 m/s was correlated with increases of 12%, 13%, and 6% in total cardiovascular events, cardiovascular mortality, and all-cause mortality, respectively.

In a vascular smooth muscle cell study, aldosterone was shown to increase collagen synthesis [46]. In an animal study, aldosterone infusion accompanied with a high sodium diet in rats was shown to cause increased arterial stiffness as evidenced by fibronectin accumulation. Moreover, this effect was independent of wall stress as shown by normotensive controls and reversal of vascular damage by treatment with an aldosterone antagonist [4]. In clinical studies, patients with PA have also been shown to have a higher PWV compared to patients with EH [5], even after adjusting for blood pressure [5,47], and this effect was reversed after adrenalectomy [6,7]. In addition, the severity of PWV has been correlated with serum aldosterone level [48]. Taken together, these studies all imply that excessive aldosterone increases arterial stiffness.

The *KCNJ5* gene is the most common site of somatic mutations in APA patients, especially in Asian countries [18,19,20,21]. *KCNJ5* mutations have been reported to increase intracellular calcium concentrations and induce activation of calcium signaling, leading to the overexpression of CYP11B2 and increase in aldosterone production [10]. APA patients with *KCNJ5* mutations have been reported to be younger, have a higher aldosterone level, lower potassium level, and higher hypertension cure rate compared to those without mutations in previous studies [15,16,17,22]. However, age, blood pressure and aldosterone level may influence the PWV in APA patients, and this may account for the diverse results reported in previous studies about the effect of *KCNJ5* mutations on PWV [23,24,26].

Our previous study revealed that APA patients with *KCNJ5* mutations had a higher left ventricular mass, and subsequently a greater regression in mass after adrenalectomy than those without mutations [25]. However, the impact of *KCNJ5* mutations on the change in PWV after adrenalectomy is still uncertain. In a study from Japan, Kitamoto et al. [23] reported a lower baseline baPWV in patients with *KCNJ5* mutations compared to those without mutations, and only patients with mutations had a significant decrease in baPWV. However, their study only enrolled a relatively small number of cases with follow-up baPWV data after adrenalectomy (33 with mutations and 5 without mutations), and subsequent data of comparisons in changes between the two groups were not available. In addition, the baseline age was younger in the patients with mutations, which may have interfered with the interpretation of lower baseline baPWV and greater change in baPWV in the patients with mutations, since younger patients generally have a lower baPWV after excluding other confounding factors. In contrast, an earlier study from our group showed that APA patients with *KCNJ5* mutations had a comparable PWV to patients without mutations both before and after matching for age, sex, and body mass index [26]. In addition, the post-operative decrease in PWV was numerically higher in the APA patients with *KCNJ5* mutations, although the difference did not reach significance (*p* = 0.106). This may have been due to the small number of enrolled patients [26].

In the present study, before PSM, the APA patients with *KCNJ5* mutations had a lower baPWV compared to those without mutations, however there was no difference after matching for age, sex, and blood pressure status. Before PSM, the patients with mutations were younger and had a shorter duration of hypertension, which may have contributed to the lower baPWV compared to those without mutations. However, after matching for age and blood pressure status, including the duration of hypertension, the difference in baPWV between the two groups diminished. This implies that a younger age and shorter hypertension duration may have accounted for the lower baPWV in the patients with *KCNJ5* mutations before PSM.

In the current study, we also found that the patients with *KCNJ5* mutations had a larger decrease in baPWV after adrenalectomy both before and after PSM compared to those without mutations. This finding was not shown in a previous study in Japan [23]. Our previous study showed a numerically higher baPWV post-operatively but without significance in APA patients with *KCNJ5* mutations comparable to those without mutations [26], and the current study confirms this finding both before and after PSM. Comparing the current study with our previous study, we enrolled more patients in the current study, which may be why the difference in baPWV reached statistical significance. The possible causes of a greater decrease in baPWV after surgery in patients with *KCNJ5* mutations include the following. First, the decreases in serum PAC level and ARR were greater in the patients with *KCNJ5* mutations than in those without mutations both before and after PSM. One previous study showed a correlation between serum aldosterone level and the severity of PWV [48]. Therefore, a greater decrease in aldosterone level after adrenalectomy may contribute to greater reversal of baPWV. Second, the rate of residual hypertension was lower in the patients with *KCNJ5* mutations after adrenalectomy. In addition, SBP (before PSM) and the number of hypertensive drugs (before and after PSM) were lower in the patients without *KCNJ5* mutations. Taken together, these findings imply better blood pressure status in the patients with *KCNJ5* mutations compared to those without mutations. The association between hypertension and arterial stiffness has been well established [49]. Therefore, this may account for the smaller reversal in baPWV after surgery in the patients without mutations. Third, in another recent study by our group, we found that the presence of *KCNJ5* mutations was associated with a lower incidence of subclinical hypercortisolism [50]. APA patients with subclinical hypercortisolism have been reported to have a higher incidence of comorbidities, including heart disease, cardiovascular events history, diabetes, and metabolic syndrome [51]. The higher incidence of subclinical hypercortisolism and subsequent comorbidity in APA patients without *KCNJ5* mutations compared to those with *KCNJ5* mutations may therefore also contribute to a smaller reversal in baPWV after surgery.

### Limitations

There are several limitations to this study. First, even though we used PSM to decrease discrepancies in age, sex, BMI, blood pressure, duration of hypertension, and number of hypertensive medications between the patients with and without *KCNJ5* mutations, unknown bias is still possible, and this may have caused an imbalance in baPWV between the two study groups. Second, we did not check somatic mutations other than *KCNJ5*, such as *ATP1A1*, *ATP2B3* [52], *CACNA1D* [53], and *CTNNB1* [54], hence we had no idea about the effects of these genes on baPWV. Third, the usage rates of ACEIs/ARBs in the APA patients with *KCNJ5* mutations were lower compared to those without mutation after matching. However, in previous studies, ACEIs or ARBs have been shown to improve arterial stiffness in patients with hypertension [55,56,57]. Therefore, the lower usage rates of ACEIs/ARBs in the patients with mutations may have caused the smaller decrease in baPWV, but this did not affect the final result of greater reversal of baPWV in patients with mutations. Fourth, since *KCNJ5* gene mutations present heterogeneity between Asian and Western populations, the results of this study may not be completely applicable to Western populations. Fifth, the use of aldosterone antagonists may influence the study results. However, the number of patients who use aldosterone antagonists was small, and it was not adequate for subgroup analysis. Sixth, we did not have the long-term follow-up data of baPWV of these patients. Whether the discrepancy of the changes of baPWV between the two groups persists or not is uncertain.

## 5. Conclusions

Compared to the APA patients without *KCNJ5* mutations, those with *KCNJ5* mutations had comparable baseline arterial stiffness but a greater regression in arterial stiffness after adrenalectomy independently of age or blood pressure.

## Figures and Tables

**Table 1:** Baseline clinical data of APA patients with and without *KCNJ5* mutations before and after PSM.

Variables	Before Propensity Score Matching			After Propensity Score Matching *		
Patient Characteristics	*KCNJ5* (+) (*n* = 126)	*KCNJ5* (−) (*n* = 87)	*p*	*KCNJ5* (+) (*n* = 66)	*KCNJ5* (−) (*n* = 66)	*p*
Age, years	47.3 ± 10.3	55.3 ± 10.5	<0.001	50.3 ± 9.6	52.5 ± 10.1	0.190
Sex, male	53(42%)	36(36%)	0.921	27(41%)	27(41%)	1.000
Height, cm	164 ± 8	163 ± 9	0.213	163 ± 8	163 ± 9	0.763
Weight, kg	66 ± 13	67 ± 14	0.774	67 ± 12	66 ± 14	0.930
Body mass index, kg/m^2^	24.5 ± 3.5	25.1 ± 3.8	0.248	24.9 ± 3.7	24.8 ± 3.7	0.914
Duration of hypertension, years	6.5 ± 6.0	9.0 ± 8.6	0.018	7.0 ± 6.1	8.4 ± 8.5	0.281
SBP, mm Hg	156 ± 22	151 ± 21	0.114	153 ± 22	152 ± 21	0.930
DBP, mm Hg	94 ± 15	89 ± 13	0.003	90 ± 14	90 ± 13	0.923
Diabetes mellitus	10(8%)	12(14%)	0.189	8(12%)	7(11%)	0.786
Dyslipidemia	21(17%)	20(23%)	0.321	15(23%)	15(23%)	1.000
Number of anti-hypertensive drugs	2.1 ± 1.1	1.9 ± 1.1	0.251	1.9 ± 1.0	2.0 ± 1.0	0.670
Hypertension medication type						
ACEI/ARB	50(40%)	44(50%)	0.172	26(39%)	38(58%)	0.037
α -Blocker	34(27%)	15(17%)	0.097	15(23%)	13(20%)	0.673
β -Blocker	44(35%)	30(34%)	0.858	20(30%)	22(33%)	0.711
CCB	93(74%)	57(65%)	0.183	45(68%)	44(67%)	0.854
Diuretics except aldosterone antagonist	8(6%)	9(10%)	0.210	3(5%)	8(12%)	0.118
Aldosterone antagonist	37(29%)	18(21%)	0.203	19(29%)	12(18%)	0.153
Vasodilator	9(7%)	6(7%)	0.963	3(5%)	4(6%)	0.700
Laboratory parameters						
Creatinine, mg/dL	0.87 ± 0.34	0.89 ± 0.29	0.694	0.89 ± 0.42	0.92 ± 0.31	0.718
Potassium, mmol/L	3.3 ± 0.6	3.8 ± 0.4	<0.001	3.3 ± 0.6	3.8 ± 0.5	<0.001
PAC ^†^, ng/dL	51(45)	34(23)	<0.001	46(42)	34(22)	0.012
PRA ^†^, ng/mL/h	0.17(0.52)	0.28(0.70)	0.171	0.17(0.45)	0.36(0.78)	0.090
ARR ^†^, ng/dL per ng/mL/h	271(737)	134(429)	0.003	254(619)	118(361)	0.017
Log PAC	1.69 ± 0.28	1.53 ± 0.27	<0.001	1.66 ± 0.29	1.55 ± 0.27	0.026
Log PRA	−0.74 ± 0.73	−0.60 ± 0.82	0.167	−0.75 ± 0.71	0.54 ± 0.89	0.133
Log ARR	2.44 ± 0.78	2.12 ± 0.80	0.005	2.41 ± 0.73	2.08 ± 0.86	0.022
baPWV ^†^, cm/s	1554(428)	1661(445)	0.088	1559(414)	1571(422)	0.831
log baPWV	3.20 ± 0.07	3.22 ± 0.08	0.046	3.21 ± 0.07	3.21 ± 0.08	0.530

Values are expressed as mean SD, median (interquartile range), or number (percentage). SBP, systolic blood pressure; DBP, diastolic blood pressure; ACEI, angiotensin-converting enzyme inhibitor; ARB, angiotensin II blocker; ARR, aldosterone–renin ratio; CCB, calcium channel blocker; PAC, plasma aldosterone concentration; PRA, plasma renin activity; ARR, aldosterone–renin activity ratio. * 1:1 matched for age, sex, BMI, SBP, DBP, duration of hypertension, and number of anti-hypertension drugs between the *KCNJ5*(+) and *KCNJ5*(−) groups. ^†^ Expressed as median and interquartile range.

**Table 2:** Changes of clinical data of APA patients with and without *KCNJ5* mutation after adrenalectomy before and after PSM.

Variables	Before Propensity Score Matching			After Propensity Score Matching *		
Patient Characteristics	*KCNJ5* (+) (*n* = 106)	*KCNJ5* (−) (*n* = 74)	*p*	*KCNJ5* (+) (*n* = 58)	*KCNJ5* (−) (*n* = 58)	*p*
∆SBP, mmHg	−26 ± 22	−16 ± 22	0.002	−24 ± 23	−16 ± 23	0.085
∆DBP, mmHg	−13 ± 16	−7 ± 13	0.003	−10 ± 14	−7 ± 14	0.142
∆Number of hypertensive drugs	−1.7 ± 1.1	−1.2 ± 1.1	0.001	−1.6 ± 1.1	−1.3 ± 1.1	0.052
∆Creatinine, mg/dL	0.19 ± 0.36	0.07 ± 0.22	0.009	0.18 ± 0.38	0.06 ± 0.23	0.427
∆Potassium, mmol/L	1.1 ± 0.7	0.4 ± 0.6	<0.001	1.1 ± 0.8	0.5 ± 0.6	<0.001
∆log PAC	−0.24 ± 0.35	−0.05 ± 0.35	<0.001	−0.19 ± 0.36	−0.04 ± 0.35	0.033
∆log PRA	0.99 ± 0.95	0.68 ± 0.99	0.034	0.99 ± 0.93	0.67 ± 1.03	0.082
∆log ARR	−1.23 ± 1.04	−0.72 ± 1.00	0.001	−1.18 ± 1.02	−0.71 ± 1.00	0.015

Values are expressed as mean SD, median (interquartile range). SBP, systolic blood pressure; DBP, diastolic blood pressure; PAC, plasma aldosterone concentration; PRA, plasma renin activity; ARR, aldosterone–renin activity ratio. * 1:1 matched for age, sex, BMI, SBP, DBP, duration of hypertension, and number of anti-hypertension drugs between the *KCNJ5*(+) and *KCNJ5*(−) groups.

**Table 3:** Paired comparisons of clinical data and pulse wave velocity data of all patients before and after adrenalectomy according to the status of *KCNJ5* mutations and the comparisons of parameters after operations between APA patients with and without mutations.

Variables	*KCNJ5* (+)			*KCNJ5* (−)			
Patient Characteristics	Baseline (*n* = 106)	Post-OP 1Y (*n* = 106)	*p*	Baseline (*n* = 74)	Post-OP 1Y (*n* = 74)	*p*	*p* ^§^
SBP, mm Hg	157 ± 22	130 ± 16	<0.001	152 ± 21	136 ± 19	<0.001	0.044
DBP, mm Hg	95 ± 15	82 ± 11	<0.001	89 ± 12	82 ± 11	<0.001	0.862
Number of hypertensive drugs	2.1 ± 1.1	0.4 ± 0.8	<0.001	1.9 ± 1.1	0.7 ± 1.0	<0.001	0.010
Creatinine, mg/dL	0.88 ± 0.36	1.07 ± 0.61	0.001	0.90 ± 0.31	0.96 ± 0.33	0.014	0.171
Potassium, mmol/L	3.3 ± 0.7	4.4 ± 0.4	<0.001	3.8 ± 0.5	4.2 ± 0.4	<0.001	0.081
Log PAC	1.71 ± 0.26	1.47 ± 0.23	<0.001	1.53 ± 0.27	1.49 ± 0.26	0.262	0.589
Log PRA	−0.74 ± 0.78	0.99 ± 0.95	<0.001	−0.55 ± 0.84	0.68 ± 0.99	<0.001	0.248
Log ARR	2.45 ± 0.82	1.21 ± 0.65	<0.001	2.08 ± 0.81	1.36 ± 0.73	<0.001	0.182
Log baPWV *	3.20 ± 0.07	3.16 ± 0.08	<0.001	3.22 ± 0.08	3.21 ± 0.09	0.127	<0.001

Values are expressed as mean SD, median (interquartile range). SBP, systolic blood pressure; DBP, diastolic blood pressure; PAC, plasma aldosterone concentration; PRA, plasma renin activity; ARR, aldosterone–renin activity ratio; baPWV, brachial–ankle pulse wave velocity. * There were 103 patients and 65 patients with and without *KCNJ5* mutations, respectively, that took a PWV exam one year after operation. ^§^ *p* value comparing the parameters after operations between APA patients with and without *KCNJ5* mutations.

**Table 4:** Paired comparison of clinical data and pulse wave velocity data of matched * patients before and after adrenalectomy according to the status of *KCNJ5* mutations.

Variables	*KCNJ5* (+)			*KCNJ5* (−)			
Patient Characteristics	Baseline (*n* = 58)	Post-OP 1Y (*n* = 58)	*p*	Baseline (*n* = 58)	Post-OP 1Y (*n* = 58)	*p*	*p* ^§^
SBP, mm Hg	154 ± 23	131 ± 15	<0.001	152 ± 20	136 ± 19	<0.001	0.092
DBP, mm Hg	92 ± 14	81 ± 10	<0.001	90 ± 12	84 ± 11	0.001	0.194
Number of hypertensive drugs	2.0 ± 1.0	0.4 ± 0.8	<0.001	2.0 ± 1.0	0.7 ± 1.0	<0.001	0.013
Creatinine, mg/dL	0.91 ± 0.45	1.09 ± 0.75	0.001	0.94 ± 0.32	1.01 ± 0.35	0.083	0.469
Potassium, mmol/L	3.3 ± 0.7	4.3 ± 0.4	<0.001	3.8 ± 0.5	4.3 ± 0.4	<0.001	0.472
Log PAC	1.68 ± 0.30	1.49 ± 0.22	<0.001	1.55 ± 0.27	1.50 ± 0.26	0.339	0.836
Log PRA	−0.75 ± 0.77	0.24 ± 0.62	<0.001	−0.52 ± 0.91	0.15 ± 0.75	<0.001	0.462
Log ARR	2.42 ± 0.79	1.24 ± 0.64	<0.001	2.07 ± 0.86	1.35 ± 0.72	<0.001	0.390
log baPWV ^†^	3.21 ± 0.06	3.17 ± 0.07	<0.001	3.22 ± 0.08	3.20 ± 0.09	0.154	0.045

Values are expressed as mean SD, median (interquartile range). SBP, systolic blood pressure; DBP, diastolic blood pressure; PAC, plasma aldosterone concentration; PRA, plasma renin activity; ARR, aldosterone–renin activity ratio; baPWV, brachial–ankle pulse wave velocity. * 1:1 matched for age, sex, BMI, SBP, DBP, duration of hypertension, and number of anti-hypertension drugs between the *KCNJ5* (+) and *KCNJ5* (−) groups. ^†^ After PSM, there were 55 patients and 52 patients with and without *KCNJ5* mutations, respectively, that took a PWV exam one year after operation. ^§^ *p* value comparing the parameters after operations between APA patients with and without *KCNJ5* mutations.

**Table 5:** Correlation of *KCNJ5* mutations with baseline log baPWV and the change of log baPWV after adrenalectomy of APA patients before and after PSM.

	Pre-PSM		Post-PSM	
Model	Pre-OP log baPWV	∆ log baPWV	Pre-OP log baPWV	∆ log baPWV
Model 1	β = −0.137, *p* = 0.046 (−0.043, 0.000)	β = −0.190, *p* = 0.014 (−0.046, −0.005)	β = −0.055, *p* = 0.530 (−0.036, 0.019)	β = −0.199, *p* = 0.040 (−0.051, −0.001)
Model 2	β= 0.065, *p* = 0.293 (−0.009, 0.029)	β= −0.194, *p* = 0.017 (−0.047, −0.005)	β= 0.004, *p* = 0.959 (−0.023, 0.024)	β= −0.191, *p* = 0.049 (−0.050, 0.000)
Model 3	β= 0.025, *p* = 0.644 (−0.013, 0.021)	β= −0.161, *p* = 0.043 (−0.043, −0.001)	β= −0.002, *p* = 0.980 (−0.021, 0.020)	β= −0.194, *p* = 0.036 (−0.049, −0.002)
Model 4	β = 0.020, *p* = 0.721 (−0.014, 0.020)	β = −0.166, *p* = 0.039 (−0.043, −0.001)	β= −0.001, *p* = 0.982 (−0.021, 0.021)	β = −0.187, *p* = 0.043 (−0.048, −0.001)

Model 1 unadjusted. Model 2 adjusted for age, sex. Model 3 adjusted for age, sex, SBP, DBP. Model 4 adjusted for age, sex, SBP, DBP, number of hypertensive drugs, hypertension duration.

## Data Availability

The data presented in this study are available on request from the corresponding author.

## Supplementary Materials

The following are available online at https://www.mdpi.com/article/10.3390/cancers13174313/s1, Table S1: Primer sequences of *KCNJ5.*

## References

[1] Funder J.W., Carey R.M., Mantero F., Murad M.H., Reincke M., Shibata H., Stowasser M., Young W.F. (2016). The management of primary aldosteronism: Case detection, diagnosis, and treatment: An endocrine society clinical practice guideline. J. Clin. Endocrinol. Metab..

[2] Käyser S.C., Dekkers T., Groenewoud H.J., van der Wilt G.J., Carel Bakx J., van der Wel M.C., Hermus A.R., Lenders J.W., Deinum J. (2016). Study heterogeneity and estimation of prevalence of primary aldosteronism: A systematic review and meta-regression analysis. J. Clin. Endocrinol. Metab..

[3] Buffolo F., Monticone S., Burrello J., Tetti M., Veglio F., Williams T.A., Mulatero P. (2017). Is primary aldosteronism still largely unrecognized?. Horm. Metab. Res..

[4] Lacolley P., Labat C., Pujol A., Delcayre C., Benetos A., Safar M. (2002). Increased carotid wall elastic modulus and fibronectin in aldosterone-salt-treated rats: Effects of eplerenone. Circulation.

[5] Strauch B., Petrák O., Wichterle D., Zelinka T., Holaj R., Widimský J. (2006). Increased arterial wall stiffness in primary aldosteronism in comparison with essential hypertension. Am. J. Hypertens..

[6] Strauch B., Petrák O., Zelinka T., Wichterle D., Holaj R., Kasalický M., Safarík L., Rosa J., Widimský J. (2008). Adrenalectomy improves arterial stiffness in primary aldosteronism. Am. J. Hypertens..

[7] Lin Y.H., Lin L.Y., Chen A., Wu X.M., Lee J.K., Su T.C., Wu V.C., Chueh S.C., Lin W.C., Lo M.T. (2012). Adrenalectomy improves increased carotid intima-media thickness and arterial stiffness in patients with aldosterone producing adenoma. Atherosclerosis.

[8] Amar L., Plouin P.F., Steichen O. (2010). Aldosterone-producing adenoma and other surgically correctable forms of primary aldosteronism. Orphanet J. Rare Dis..

[9] Rossi G.P., Di Bello V., Ganzaroli C., Sacchetto A., Cesari M., Bertini A., Giorgi D., Scognamiglio R., Mariani M., Pessina A.C. (2002). Excess ldosterone is associated with alterations of myocardial texture in primary aldosteronism. Hypertension.

[10] Choi M., Scholl U.I., Yue P., Bjorklund P., Zhao B., Nelson-Williams C., Ji W., Cho Y., Patel A., Men C.J. (2011). K+ channel mutations in adrenal aldosterone-producing adenomas and hereditary hypertension. Science.

[11] El Zein R.M., Boulkroun S., Fernandes-Rosa F.L., Zennaro M.C. (2018). Molecular genetics of conn adenomas in the era of exome analysis. Presse Med..

[12] De Sousa K., Boulkroun S., Baron S., Nanba K., Wack M., Rainey W.E., Rocha A., Giscos-Douriez I., Meatchi T., Amar L. (2020). Genetic, cellular, and molecular heterogeneity in adrenals with aldosterone-producing adenoma. Hypertension.

[13] Nanba K., Omata K., Gomez-Sanchez C.E., Stratakis C.A., Demidowich A.P., Suzuki M., Thompson L.D.R., Cohen D.L., Luther J.M., Gellert L. (2019). Genetic characteristics of aldosterone-producing adenomas in blacks. Hypertension.

[14] Nanba K., Omata K., Else T., Beck P.C.C., Nanba A.T., Turcu A.F., Miller B.S., Giordano T.J., Tomlins S.A., Rainey W.E. (2018). Targeted molecular characterization of aldosterone-producing adenomas in white americans. J. Clin. Endocrinol. Metab..

[15] Fernandes-Rosa F.L., Williams T.A., Riester A., Steichen O., Beuschlein F., Boulkroun S., Strom T.M., Monticone S., Amar L., Meatchi T. (2014). Genetic spectrum and clinical correlates of somatic mutations in aldosterone-producing adenoma. Hypertension.

[16] Boulkroun S., Beuschlein F., Rossi G.P., Golib-Dzib J.F., Fischer E., Amar L., Mulatero P., Samson-Couterie B., Hahner S., Quinkler M. (2012). Prevalence, clinical, and molecular correlates of kcnj5 mutations in primary aldosteronism. Hypertension.

[17] Lenzini L., Rossitto G., Maiolino G., Letizia C., Funder J.W., Rossi G.P. (2015). A meta-analysis of somatic kcnj5 k(+) channel mutations in 1636 patients with an aldosterone-producing adenoma. J. Clin. Endocrinol. Metab..

[18] Wu V.C., Wang S.M., Chueh S.J., Yang S.Y., Huang K.H., Lin Y.H., Wang J.J., Connolly R., Hu Y.H., Gomez-Sanchez C.E. (2017). The prevalence of ctnnb1 mutations in primary aldosteronism and consequences for clinical outcomes. Sci. Rep..

[19] Taguchi R., Yamada M., Nakajima Y., Satoh T., Hashimoto K., Shibusawa N., Ozawa A., Okada S., Rokutanda N., Takata D. (2012). Expression and mutations of kcnj5 mrna in japanese patients with aldosterone-producing adenomas. J. Clin. Endocrinol. Metab..

[20] Zheng F.-F., Zhu L.-M., Nie A.-F., Li X.-Y., Lin J.-R., Zhang K., Chen J., Zhou W.-L., Shen Z.-J., Zhu Y.-C. (2015). Clinical characteristics of somatic mutations in chinese patients with aldosterone-producing adenoma. Hypertension.

[21] Hong A.R., Kim J.H., Song Y.S., Lee K.E., Seo S.H., Seong M.-W., Shin C.S., Kim S.W., Kim S.Y. (2016). Genetics of aldosterone-producing adenoma in korean patients. PLoS ONE.

[22] Rossi G.P., Cesari M., Letizia C., Seccia T.M., Cicala M.V., Zinnamosca L., Kuppusamy M., Mareso S., Sciomer S., Iacobone M. (2014). Kcnj5 gene somatic mutations affect cardiac remodelling but do not preclude cure of high blood pressure and regression of left ventricular hypertrophy in primary aldosteronism. J. Hypertens..

[23] Kitamoto T., Suematsu S., Matsuzawa Y., Saito J., Omura M., Nishikawa T. (2015). Comparison of cardiovascular complications in patients with and without kcnj5 gene mutations harboring aldosterone-producing adenomas. J. Atheroscler. Thromb..

[24] Wu V.C., Huang K.H., Peng K.Y., Tsai Y.C., Wu C.H., Wang S.M., Yang S.Y., Lin L.Y., Chang C.C., Lin Y.H. (2015). Prevalence and clinical correlates of somatic mutation in aldosterone producing adenoma-taiwanese population. Sci. Rep..

[25] Chang Y.Y., Tsai C.H., Peng S.Y., Chen Z.W., Chang C.C., Lee B.C., Liao C.W., Pan C.T., Chen Y.L., Lin L.C. (2021). Kcnj5 somatic mutations in aldosterone-producing adenoma are associated with a worse baseline status and better recovery of left ventricular remodeling and diastolic function. Hypertension.

[26] Chang C.H., Hu Y.H., Tsai Y.C., Wu C.H., Wang S.M., Lin L.Y., Lin Y.H., Satoh F., Wu K.D., Wu V.C. (2017). Arterial stiffness and blood pressure improvement in aldosterone-producing adenoma harboring kcnj5 mutations after adrenalectomy. Oncotarget.

[27] Williams T.A., Lenders J.W.M., Mulatero P., Burrello J., Rottenkolber M., Adolf C., Satoh F., Amar L., Quinkler M., Deinum J. (2017). Outcomes after adrenalectomy for unilateral primary aldosteronism: An international consensus on outcome measures and analysis of remission rates in an international cohort. Lancet Diabetes Endocrinol..

[28] Wu V.C., Hu Y.H., Er L.K., Yen R.F., Chang C.H., Chang Y.L., Lu C.C., Chang C.C., Lin J.H., Lin Y.H. (2017). Case detection and diagnosis of primary aldosteronism—the consensus of taiwan society of aldosteronism. J. Formos. Med. Assoc..

[29] Rossi G.P., Belfiore A., Bernini G., Desideri G., Fabris B., Ferri C., Giacchetti G., Letizia C., Maccario M., Mallamaci F. (2007). Comparison of the captopril and the saline infusion test for excluding aldosterone-producing adenoma. Hypertension.

[30] Wu V.C., Chang H.W., Liu K.L., Lin Y.H., Chueh S.C., Lin W.C., Ho Y.L., Huang J.W., Chiang C.K., Yang S.Y. (2009). Primary aldosteronism: Diagnostic accuracy of the losartan and captopril tests. Am. J. Hypertens..

[31] Wu V.C., Yang S.Y., Lin J.W., Cheng B.W., Kuo C.C., Tsai C.T., Chu T.S., Huang K.H., Wang S.M., Lin Y.H. (2011). Kidney impairment in primary aldosteronism. Clin. Chim. Acta.

[32] Schwartz G.L., Turner S.T. (2005). Screening for primary aldosteronism in essential hypertension: Diagnostic accuracy of the ratio of plasma aldosterone concentration to plasma renin activity. Clin. Chem..

[33] Chao C.T., Wu V.C., Kuo C.C., Lin Y.H., Chang C.C., Chueh S.J., Wu K.D., Pimenta E., Stowasser M. (2013). Diagnosis and management of primary aldosteronism: An updated review. Ann. Med..

[34] McDonald D.A. (1968). Regional pulse-wave velocity in the arterial tree. J. Appl. Physiol..

[35] Nomura K., Toraya S., Horiba N., Ujihara M., Aiba M., Demura H. (1992). Plasma aldosterone response to upright posture and angiotensin ii infusion in aldosterone-producing adenoma. J. Clin. Endocrinol. Metab..

[36] Novitsky Y.W., Kercher K.W., Rosen M.J., Cobb W.S., Jyothinagaram S., Heniford B.T. (2005). Clinical outcomes of laparoscopic adrenalectomy for lateralizing nodular hyperplasia. Surgery.

[37] Azizan E.A.B., Murthy M., Stowasser M., Gordon R., Kowalski B., Xu S., Brown M.J., O’Shaughnessy K.M. (2012). Somatic mutations affecting the selectivity filter of kcnj5 are frequent in 2 large unselected collections of adrenal aldosteronomas. Hypertension.

[38] Laurent S., Boutouyrie P., Asmar R., Gautier I., Laloux B., Guize L., Ducimetiere P., Benetos A. (2001). Aortic stiffness is an independent predictor of all-cause and cardiovascular mortality in hypertensive patients. Hypertension.

[39] Munakata M. (2014). Brachial-ankle pulse wave velocity in the measurement of arterial stiffness: Recent evidence and clinical applications. Curr. Hypertens. Rev..

[40] Cortez-Cooper M.Y., Supak J.A., Tanaka H. (2003). A new device for automatic measurements of arterial stiffness and ankle-brachial index. Am. J. Cardiol..

[41] Wang J.W., Zhou Z.Q., Hu D.Y. (2012). Prevalence of arterial stiffness in north china, and associations with risk factors of cardiovascular disease: A community-based study. BMC Cardiovasc. Disord..

[42] Ninomiya T., Kojima I., Doi Y., Fukuhara M., Hirakawa Y., Hata J., Kitazono T., Kiyohara Y. (2013). Brachial-ankle pulse wave velocity predicts the development of cardiovascular disease in a general japanese population: The hisayama study. J. Hypertens..

[43] Vlachopoulos C., Aznaouridis K., Terentes-Printzios D., Ioakeimidis N., Stefanadis C. (2012). Prediction of cardiovascular events and all-cause mortality with brachial-ankle elasticity index: A systematic review and meta-analysis. Hypertension.

[44] Sheng C.S., Li Y., Li L.H., Huang Q.F., Zeng W.F., Kang Y.Y., Zhang L., Liu M., Wei F.F., Li G.L. (2014). Brachial-ankle pulse wave velocity as a predictor of mortality in elderly chinese. Hypertension.

[45] Snijder M.B., Stronks K., Agyemang C., Busschers W.B., Peters R.J., van den Born B.J. (2015). Ethnic differences in arterial stiffness the helius study. Int. J. Cardiol..

[46] Callera G.E., Touyz R.M., Tostes R.C., Yogi A., He Y., Malkinson S., Schiffrin E.L. (2005). Aldosterone activates vascular p38map kinase and nadph oxidase via c-src. Hypertension.

[47] Ambrosino P., Lupoli R., Tortora A., Cacciapuoti M., Lupoli G.A., Tarantino P., Nasto A., Di Minno M.N. (2016). Cardiovascular risk markers in patients with primary aldosteronism: A systematic review and meta-analysis of literature studies. Int. J. Cardiol..

[48] Park S., Kim J.B., Shim C.Y., Ko Y.G., Choi D., Jang Y., Chung N. (2007). The influence of serum aldosterone and the aldosterone-renin ratio on pulse wave velocity in hypertensive patients. J. Hypertens..

[49] Cecelja M., Chowienczyk P. (2009). Dissociation of aortic pulse wave velocity with risk factors for cardiovascular disease other than hypertension: A systematic review. Hypertension.

[50] Peng K.Y., Liao H.W., Chan C.K., Lin W.C., Yang S.Y., Tsai Y.C., Huang K.H., Lin Y.H., Chueh J.S., Wu V.C. (2020). Presence of subclinical hypercortisolism in clinical aldosterone-producing adenomas predicts lower clinical success. Hypertension.

[51] Tang L., Li X., Wang B., Ma X., Li H., Gao Y., Gu L., Nie W., Zhang X. (2018). Clinical characteristics of aldosterone- and cortisol-coproducing adrenal adenoma in primary aldosteronism. Int. J. Endocrinol..

[52] Beuschlein F., Boulkroun S., Osswald A., Wieland T., Nielsen H.N., Lichtenauer U.D., Penton D., Schack V.R., Amar L., Fischer E. (2013). Somatic mutations in atp1a1 and atp2b3 lead to aldosterone-producing adenomas and secondary hypertension. Nat. Genet..

[53] Scholl U.I., Goh G., Stolting G., de Oliveira R.C., Choi M., Overton J.D., Fonseca A.L., Korah R., Starker L.F., Kunstman J.W. (2013). Somatic and germline cacna1d calcium channel mutations in aldosterone-producing adenomas and primary aldosteronism. Nat. Genet..

[54] Scholl U.I., Healy J.M., Thiel A., Fonseca A.L., Brown T.C., Kunstman J.W., Horne M.J., Dietrich D., Riemer J., Kücükköylü S. (2015). Novel somatic mutations in primary hyperaldosteronism are related to the clinical, radiological and pathological phenotype. Clin. Endocrinol..

[55] Shahin Y., Khan J.A., Chetter I. (2012). Angiotensin converting enzyme inhibitors effect on arterial stiffness and wave reflections: A meta-analysis and meta-regression of randomised controlled trials. Atherosclerosis.

[56] Frimodt-Møller M., Kamper A.L., Strandgaard S., Kreiner S., Nielsen A.H. (2012). Beneficial effects on arterial stiffness and pulse-wave reflection of combined enalapril and candesartan in chronic kidney disease—A randomized trial. PLoS ONE.

[57] Anan F., Takahashi N., Ooie T., Yufu K., Hara M., Nakagawa M., Yonemochi H., Saikawa T., Yoshimatsu H. (2005). Effects of valsartan and perindopril combination therapy on left ventricular hypertrophy and aortic arterial stiffness in patients with essential hypertension. Eur. J. Clin. Pharmacol..

