# Supplementary material for: KCNJ5 Somatic Mutations in Aldosterone-Producing Adenoma Are Associated with a Greater Recovery of Arterial Stiffness
Source: Cancers (Basel). 2021 Aug 26;13(17):4313. doi: 10.3390/cancers13174313 (PMC8431463; doi:10.3390/cancers13174313)
Supplement: Supplementary file 1 [file cancers-13-04313-s001.zip › cancers-1335641-supplementary.pdf]

cancers

MDPI

Supplementary Material: *KCNJ5* Somatic Mutations in Aldosterone-Producing Adenoma are associated with a greater recovery of arterial stiffness

Yi-Yao Chang, Chien-Ting Pan, Zheng-Wei Chen, Cheng-Hsuan Tsai, Shih-Yuan Peng, Chin-Chen Chang, Bo-Ching Lee, Che-Wei Liao, Kang-Yung Peng, Yu-Wei Chiu, Chia-Hung Chou, Vin-Cent Wu, Li-Yu Daisy Liu, Chi-Sheng Hung and Yen-Hung Lin

Table S1. Primer sequences of *KCNJ5*.

Primer name	Primer sequence (5'→3')
*KCNJ5*_1F	GATGGTGTCTTTTTAACTCAAAGC
*KCNJ5*_1R	GTGATGACTCGGAAGCCATACC
*KCNJ5*_2F	CTTTCCTGTTCTCCATTGAGACC
*KCNJ5*_2R	CTGAGGAGGACAAAGCGCC
*KCNJ5*_3F	ATGCATGTAACTTCCGTTTCCC
*KCNJ5*_3R	GCCAGTGACAGGAGGTCTTAGG
*KCNJ5*_4F	CTTCATTTGGTGGCTCATTGC
*KCNJ5*_4R	GGGACTTGATGAGCTTGGC
